# Halotolerant *Bacillus altitudinis* WR10 improves salt tolerance in wheat *via* a multi-level mechanism

**DOI:** 10.3389/fpls.2022.941388

**Published:** 2022-07-14

**Authors:** Zonghao Yue, Yanjuan Chen, Yifan Wang, Limin Zheng, Qiaoyang Zhang, Yongchuang Liu, Chunhong Hu, Can Chen, Keshi Ma, Zhongke Sun

**Affiliations:** ^1^College of Life Sciences and Agronomy, Zhoukou Normal University, Zhoukou, China; ^2^School of Mechanical and Electrical Engineering, Zhoukou Normal University, Zhoukou, China; ^3^College of Biological Engineering, Henan University of Technology, Zhengzhou, China

**Keywords:** *Bacillus*, salt stress, wheat, antioxidant enzyme, hydrogen peroxide, glutathione, phenylpropanoid biosynthesis

## Abstract

Soil salinity is an important abiotic stress factor that seriously affects the crop growth and yield. Use of plant-derived microorganisms is a promising strategy to alleviate salt stress. In a previous study, the endophytic strain *Bacillus altitudinis* WR10 isolated from wheat roots showed high salt resistance. In this study, we investigated the efficacy of WR10 in improving the salt tolerance of wheat and its potential mechanisms using a hydroponic test. Under salt stress, WR10 inoculation significantly increased the lengths and dry weights of the roots and shoots, indicating that WR10 improves wheat salt tolerance at the seedling stage. WR10 inoculation significantly reduced Na^+^ accumulation and enhanced K^+^, P, and Ca^2+^ uptake in salt-stressed plants, which can be attributed to the upregulated gene expression of H^+^-ATPase as well as the P-solubilizing and biofilm-producing characteristics of WR10. At the transcriptional level, L-ascorbate peroxidase (APX), glutathione (GSH) synthetase related to GSH biosynthesis, and phenylpropanoid biosynthesis genes (CYP73A, 4CL, and CAD) were significantly upregulated, whereas those of GSH metabolism genes (glutathione S-transferase and gamma-glutamyltranspeptidase) were significantly downregulated in WR10-applied wheat roots under salt stress. These changes increased the APX activity and GSH levels and resulted in a decrease in hydrogen peroxide levels. Additionally, a decrease in proline content was observed in WR10-inoculated plants under salt stress because of WR10-induced upregulation of proline dehydrogenase gene expression. These results provide supporting evidence that WR10 improves wheat salt tolerance via more than one mechanism and open a window of opportunity for WR10 application in salinized soil.

## Introduction

Soil salinization has become a serious global problem owing to climate change, seawater intrusion, improper irrigation, and other factors. More than 833 million hectares of soil and 10% of farmland worldwide are under the threat of salinization (Food and Agriculture Organization [FAO], 2021). By 2050, the salinity of farmlands is suggested to increase to 50% (Shrivastava and Kumar, 2015). High salinity induces ionic toxicity, osmotic stress, oxidative stress, and nutrient depletion, which seriously endanger the crop growth and decrease the crop yield (Muranaka et al., 2002; Lodeyro and Carrillo, 2015; Zhu, 2016). The annual economic loss caused by salt stress-induced crop yield exceeds 27.3 billion dollars (Qadir et al., 2014). Therefore, salt stress poses a major challenge to global food security. With the rapid increase in world population (estimated to reach 9.1 billion in 2050) and food demand (estimated to increase by 70%), this challenge will become more severe (Food and Agriculture Organization [FAO], 2009). Therefore, improving crop salt tolerance and alleviating the adverse effects of salt stress are paramount for sustainable agricultural development and global food security.

Wheat (*Triticum aestivum* L.) is an important cereal crop that showed 760.92 million tonnes global production in 2020. It is the source of approximately 20% of total dietary calories and proteins worldwide (Shiferaw et al., 2013). Most wheat cultivars are moderately tolerant or sensitive to salt stress (Quan et al., 2021). When wheat plants are grown in soil with 8–10 dS/m electrical conductivity (moderate salinity), 20–28% yield loss is observed (Satir and Berberoglu, 2016). Substantial efforts to develop salt-tolerant wheat varieties via conventional breeding and genetic engineering methods have been made during the last few decades, but only limited success has been achieved because of the time constraints, intricate procedures, and ethical issues (Ashraf and Akram, 2009).

A large number of plant growth promoting bacteria (PGPB) are found in the rhizosphere and roots of plants. Many PGPB belonging to the genera *Bacillus*, *Halomonas*, *Enterobacter*, *Moraxella*, *Pseudomonas*, *Zhihengliuella*, *Staphylococcus*, *Oceanobacillus*, *Thalassobacillus*, *Halobacillus*, *Dietzia*, *Marinibacillus*, *Planococcus*, and *Promicromonospora* sp. are tolerant to high salt conditions and play important roles in improving plant salt tolerance (Raheem and Ali, 2015; Bharti et al., 2016; Orhan, 2016; Mukherjee et al., 2019; Orhan and Demirci, 2020). For example, *Bacillus subtilis* BERA 71 enhances plant biomass and synthesis of photosynthetic pigments in chickpea exposed to saline conditions (Abd_Allah et al., 2018). Inoculation with *Hallobacillus* sp. SL3 and *Bacillus halodentinicans* PU62 significantly increases the root length and dry weight of wheat seedlings under salt stress (Ramadoss et al., 2013). *Pseudomonas* sp. UW3 + UW and *Pseudomonas corrugate* CMH3 significantly increase the stem biomass of oats grown in saline field soils (Chang et al., 2014). Hence, use of PGPB may be a desirable strategy to mitigate the adverse effects of soil salinity.

In a previous study, we isolated a wheat endophytic PGPB strain *Bacillus altitudinis* WR10. It shows many plant growth-promoting traits, such as ACC deaminase production, indole-3-acetic acid secretion, and phosphate solubilization, and improves the ability of the host to withstand numerous abiotic stresses (Sun et al., 2017; Yue et al., 2019, 2021). WR10 is a halotolerant strain that tolerates up to 12% NaCl and increases seed germination of wheat under salt stress (Yue et al., 2019). Due to its inherent tolerance to NaCl, multiple plant growth-promoting traits, and intimate interaction with wheat, we speculated that WR10 may affect plant salt stress tolerance.

Therefore, this study aimed to evaluate the efficacy of WR10 in alleviating salt stress by determining the lengths and dry weights of the roots and shoots of wheat. PGPB can use multiple mechanisms, including production of phytohormones, regulation of Na^+^ homeostasis, improved nutrient uptake, increased number of active oxygen scavengers, and osmolyte accumulation, to improve plant tolerance (Qin et al., 2016; Mishra et al., 2021). We hypothesized that the salinity-alleviating property of WR10 could be attributed to one or more of these mechanisms. To further clarify how WR10 interacts with its wheat host, root transcriptome and biochemical experiments (including proline, nutrients, and antioxidant compounds) were performed. This study provides a theoretical basis for the application of WR10 in wheat under salt stress.

## Materials and methods

### Seed germination, bacterial culture, and preparation of bacterial suspension

Seeds of Zhoumai 36, a new variety cultivated by the Zhoukou Academy of Agricultural Sciences (Zhoukou, China), were surface-sterilized for 15 min using 5% sodium hypochlorite. After washing thrice with sterile deionized water, the seeds were placed in a plastic basin (21.3 cm × 16.3 cm × 6.8 cm) with sterile water and germinated for two days in the dark at 25°C.

*Bacillus altitudinis* WR10 preserved in 30% glycerol was inoculated into 5 mL of sterile Luria-Bertani (LB) broth and cultured for 24 h at 37°C and 150 rpm. Then, 1 mL of the bacterial suspension was added to 100 mL of LB broth and cultured for another 24 h under the same conditions. Finally, the bacterial suspension was centrifuged for 10 min at 9,000 × *g* and the bacterial pellet was collected. After washing twice with sterile deionized water, the pellet was resuspended in sterile deionized water to prepare a bacterial suspension with a final concentration of 3 × 10^10^ cfu/mL.

### Effect of WR10 on wheat growth under NaCl stress condition

A total of 240 seeds with consistent germination were selected and randomly assigned to four groups: the control group without NaCl and WR10, WR10 group with WR10 and without NaCl, NaCl group with NaCl and without WR10, and WR10 + NaCl group with NaCl and WR10. Each treatment group contained 60 seeds. The seeds were placed in glass beakers containing 600 mL of 1/2 Hoagland’s medium with or without 150 mmol/L NaCl and 3 × 10^7^ cfu/mL of WR10. NaCl concentration was selected based on a salt tolerance experiment by Zhoumai 36 (Supplementary Figure 1). To avoid osmotic shock in wheat seedlings, salinity was increased gradually in increments of 50 mmol/L every two days to reach a final salt concentration of 150 mmol/L. In groups inoculated with WR10, 0.6 mL bacterial suspension was added to each beaker, whereas groups without WR10 were supplemented with an equal volume of sterile solution. After two weeks of growth at 22 ± 1°C, 60% relative humidity, and a photoperiod of 14 h light/10 h dark, all wheat seedlings were harvested. During plant growth, the culture solutions were renewed every two days. Some seedlings were randomly selected to measure the root and shoot lengths. Then, these seedlings were placed in an oven and dried for 72 h at 75°C. The dried roots and shoots were cut off to determine the dry weight and element content. The roots and shoots of other seedlings were separated; the shoots were used for proline detection and the roots were used for transcriptome sequencing after quick freezing in liquid nitrogen.

### Determination of proline content

The proline content was determined using a proline colorimetric assay kit (Elabscience Biotechnology Co., Ltd., Wuhan, China). Briefly, 0.1 g of fresh shoot was homogenized with 1 mL of extracting solution from the kit and centrifuged at 10,000 × *g* for 15 min. The supernatant was collected and the proline content was determined according to the manufacturer’s instructions.

### Measurement of elements in wheat tissues

Dry roots or shoots were digested as described in our previous study (Sun et al., 2021). For potassium (K) and phosphorus (P) determination, 0.1 g of ground tissues were digested using 5 mL H_2_SO_4_ and 2 mL H_2_O_2_, and the digestion solutions were then diluted with 20 mL deionized water and neutralized with 10 M NaOH. For sodium (Na), calcium (Ca), and magnesium (Mg) determination, 0.1 g of ground tissues were extracted for 2 h by adding 20 mL of 0.5 mol/L HNO_3_ at 37°C and 200 rpm. The extracted solutions were centrifuged at 10,000 × *g* for 5 min and the supernatants were collected. Na content was measured using flame atomic absorption spectrophotometry (A3AFG, Persee, Beijing, China). K, P, Ca and Mg contents were determined using commercial biochemical assay kits (Elabscience, China). All absorbance measurements were performed on a SpectraMax i3x microplate reader (Molecular Devices, Sunnyvale, CA, United States).

### Root RNA isolation and sequencing

Total RNA was extracted from root samples using the PureLink Plant RNA reagent (Invitrogen, United States), according to the manufacturer’s protocol. RNA concentration was measured using a Nanodrop 2000c ultra-micro spectrophotometer (Thermo Fisher, Waltham, MA, United States). RNA integrity was assessed via 1% agarose gel electrophoresis using an Agilent Bioanalyzer 2100 (Agilent Technologies, Santa Clara, CA, United States). High-quality RNA was used to construct cDNA libraries using the TruSeq RNA Sample Prep Kit (Illumina, San Diego, CA, United States). Sequencing was performed using the Illumina Novaseq 6000 system (Illumina, United States) by Majorbio Biotech Co., Ltd. (Shanghai, China).

Raw reads were filtered to remove the adapter, low-quality, higher N-ratio (>10%), and <50 bp reads, and clean reads were obtained. Using the TopHat2 software, high-quality clean reads were aligned with the wheat reference genome (version IWGSC). The resulting mapped reads were further assembled using the StringTie software. Functional annotation of genes or transcripts was performed using the NR, Swiss-Prot, Pfam, EggNOG, GO, and KEGG databases. All raw data were submitted to the NCBI Sequence Read Archive under the accession numbers SRR17951558-SRR17951569.

### Differentially expressed gene screening and enrichment analysis

The expression levels of genes were analyzed via the transcripts per million reads (TPM) method using the RSEM software. Differential gene expression between different treatment groups was analyzed using the DESeq2 software. The screening criterion for differentially expressed genes (DEGs) was *p* value adjusted with Benjamini/Hochberg’s approach <0.05, and | log2 (fold change, FC)| ≥ 1. GO and KEGG enrichment analyses for DEGs were performed using the Goatools and KOBAS software, respectively. Using Venny 2.1 software, a Venn diagram was produced. Using the XLSTAT software, principal component analysis (PCA) was conducted to assess the relationship between biological repetitions in all groups.

### Measurement of hydrogen peroxide levels, L-ascorbate peroxidase activity, peroxidase activity, and glutathione levels

Hydrogen peroxide (H_2_O_2_) levels, L-ascorbate peroxidase (APX) activity, and peroxidase (POD) activity in roots, and glutathione (GSH) levels in roots and shoots were measured using colorimetric assay kits (Elabscience, China), according to the manufacturer’s instructions. H_2_O_2_ levels, APX activity, POD activity, and GSH levels were expressed as mmoL/g FW, U/g FW, U/mg protein, and μmoL/mg protein, respectively.

### Statistical analysis

Data were expressed as the mean ± standard deviation (SD). Before testing for differences between the two groups (Control vs. NaCl; NaCl vs. WR10 + NaCl), tests of normality (Kolmogorov–Smirnov) and equal variance (Levene median) were performed. Student’s *t*-test (parametric) was used when the conditions of normality and equal variance were met. When normality test failed or unequal variances were detected, the Mann-Whitney rank sum test (non-parametric) was used. All statistical analyses were performed using IBM SPSS Statistics 19.0 (IBM, Armonk, NY, United States). *p* < 0.05 was considered to be statistically significant.

## Results

### *Bacillus altitudinis* WR10 improves wheat salt tolerance

**FIGURE 1:**
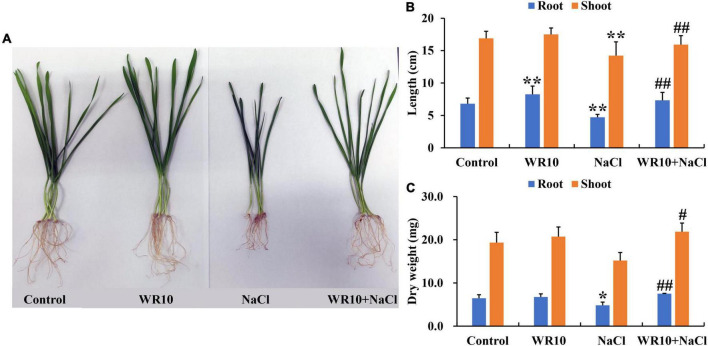
*Bacillus altitudinis* WR10 alleviates the adverse effects of salt stress on wheat seedlings. **(A)** Photograph of wheat seedlings; **(B)** lengths of roots and shoots; **(C)** dry weights of roots and shoots. Control, without NaCl and WR10; WR10, with WR10 and without NaCl; NaCl, with NaCl and without WR10; WR10 + NaCl, with NaCl and WR10. Data are expressed as the means ± SD (*n* = 12). *p < 0.05, **p < 0.01 (vs. control); ^#^*p* < 0.05, ^##^*p* < 0.01 (vs. NaCl).

Compared to the control group, salt stress significantly inhibited the growth of wheat seedlings, whereas inoculation with WR10 alleviated the damage caused by salt stress in wheat (Figure 1A). Specifically, WR10 inoculation significantly increased the root length, shoot length, root dry weight, and shoot dry weight by 1. 56-, 1. 12-, 1. 56-, and 1.44-fold, respectively, compared to the WR10 uninoculated group under salt stress (Figures 1B–C).

### *Bacillus altitudinis* WR10 reduces Na^+^ accumulation and promotes nutrient absorption in wheat seedlings under salt stress

**FIGURE 2:**
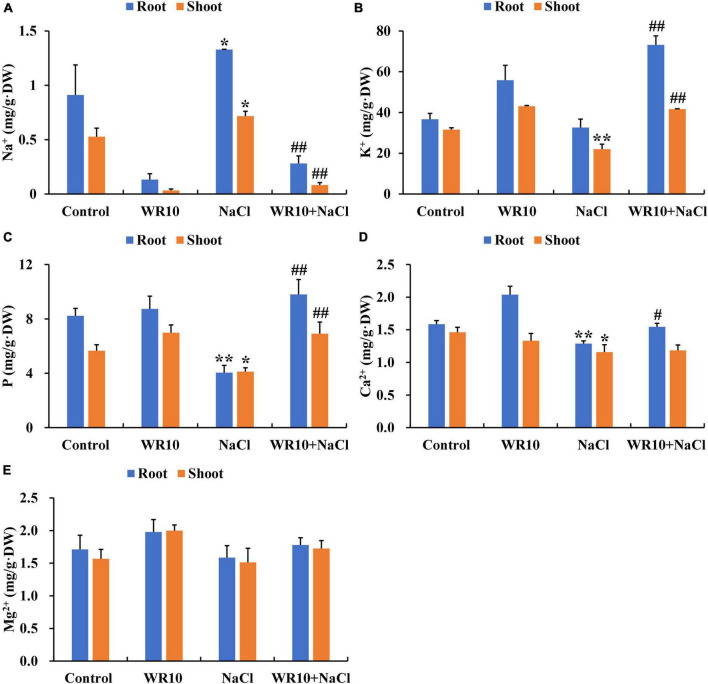
Effects of *B. altitudinis* WR10 on the levels of Na^+^ **(A)**, K^+^ **(B)**, P **(C)**, Ca^2+^ **(D)**, and Mg^2+^ **(E)** in the roots and shoots of wheat seedlings under salt stress. Control, without NaCl and WR10; WR10, with WR10 and without NaCl; NaCl, with NaCl and without WR10; WR10 + NaCl, with NaCl and WR10. Data are expressed as the means ± SD (*n* = 6). *p < 0.05, **p < 0.01 (vs. control); ^#^*p* < 0.05, ^##^*p* < 0.01 (vs. NaCl).

Compared to the control group, Na^+^ content of roots and shoots in salt-stressed wheat seedlings significantly increased by 1.46- and 1.36-fold, respectively (Figure 2A). In addition, salt stress significantly decreased the K^+^ content in shoots by 0.30-fold, P content in roots and shoots by 0.51- and 0.27-fold, respectively, and Ca^2+^ content in roots and shoots by 0.19- and 0.21-fold, respectively (Figures 2B–D). In contrast, WR10 inoculation significantly reduced Na^+^ content in roots and shoots by 0.79- and 0.89-fold, respectively, but increased K^+^ content in roots and shoots by 2.24- and 1.89-fold, P content in roots and shoots by 2.42- and 1.68-fold, and Ca^2+^ content in roots by 1.20-fold, respectively, compared to the WR10 uninoculated group under salt stress (Figures 2B–D). In addition, the content of Mg^2+^ did not change significantly in any treatment group (Figure 2E).

### *Bacillus altitudinis* WR10 alters the gene expression levels in wheat roots under salt stress

RNA-sequencing (RNA-seq) of 12 root samples produced 807.6 million raw reads. After quality control, a total of 798.7 million clean reads were obtained and mapped to the wheat reference genome with 78.75–83.22% mapped rate (Supplementary Tables 1, 2). These mapped reads were assembled into 86781 genes, 99.72% of which were successfully annotated (Supplementary Table 3).

**FIGURE 3:**
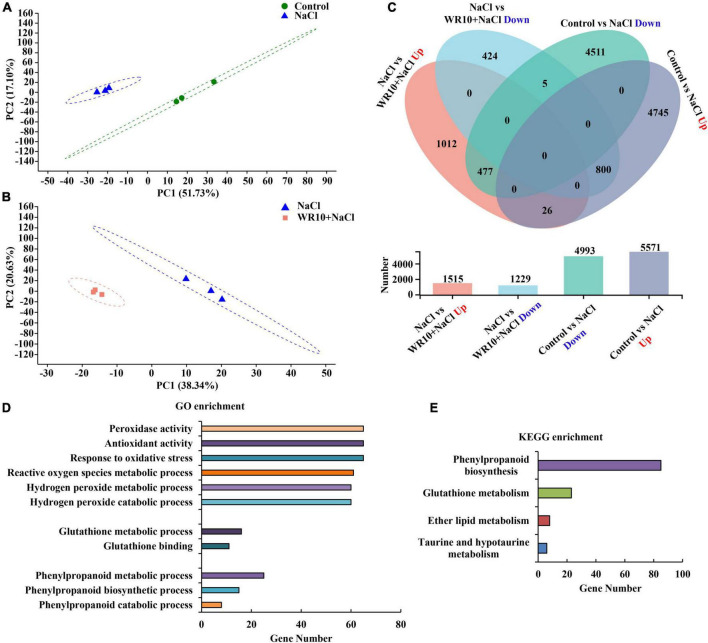
RNA-sequencing analyses of wheat roots. **(A)** Principal component analysis (PCA) analyses of Control and NaCl groups. **(B)** PCA analyses of NaCl and WR10 + NaCl groups. **(C)** Venn diagram displaying the differentially expressed genes (DEGs) in Control vs. NaCl and NaCl vs. WR10 + NaCl. **(D,E)** GO and KEGG enrichment analyses of DEGs in NaCl vs WR10 + NaCl. Control, without NaCl and WR10; WR10, with WR10 and without NaCl; NaCl, with NaCl and without WR10; WR10 + NaCl, with NaCl and WR10.

PCA analysis showed that the samples of the control and NaCl groups and NaCl and NaCl + WR10 groups significantly separated from each other, indicating different gene expression patterns (Figures 3A–B). Further differential expression analysis revealed a total of 10,563 genes differentially expressed in NaCl-treated wheat compared with the control group, of which 5,571 genes were significantly upregulated and 4,993 genes were significantly downregulated. Moreover, 2744 DEGs were observed between NaCl and NaCl + WR10 groups. Among these DEGs, 1,515 were significantly upregulated and 1,229 were significantly downregulated in the NaCl + WR10 group (Figure 3C). Using GO enrichment analysis, DEGs in NaCl vs NaCl + WR10 were significantly enriched into 239 GO terms, including “peroxidase (POD) activity” (GO:0004601), “antioxidant activity” (GO:0016209), “response to oxidative stress” (GO:0006979), “reactive oxygen species (ROS) metabolic process” (GO:0072593), “H_2_O_2_ catabolic process” (GO:0042744), “H_2_O_2_ metabolic process” (GO:0042743), “GSH metabolic process” (GO:0006749), “GSH binding” (GO:0043295), “phenylpropanoid metabolic process” (GO:0009698), “phenylpropanoid biosynthetic process” (GO:0009699), and “phenylpropanoid catabolic process” (GO:0046271) (Figure 3D). Moreover, KEGG enrichment analysis showed that DEGs in NaCl vs NaCl + WR10 were significantly enriched in four pathways, in which “phenylpropanoid biosynthesis” (map00940) and “GSH metabolism” had the most DEGs (Figure 3E).

### *Bacillus altitudinis* WR10 increases APX activity and reduces H_2_O_2_ levels under salt stress

**FIGURE 4:**
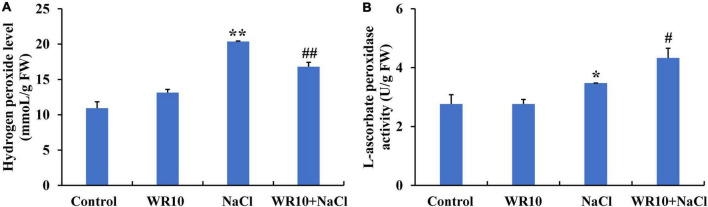
*Bacillus altitudinis* WR10 decreases H_2_O_2_ levels and increases APX activity in the roots of wheat seedlings under salt stress. **(A)** H_2_O_2_ levels in the roots of wheat seedlings; **(B)** APX activity in the roots of wheat seedlings. Control, without NaCl and WR10; WR10, with WR10 and without NaCl; NaCl, with NaCl and without WR10; WR10 + NaCl, with NaCl and WR10. Data are expressed as the means ± SD (*n* = 6). *p < 0.05, **p < 0.01 (vs. control); ^#^*p* < 0.05, ^##^*p* < 0.01 (vs. NaCl).

Interestingly, 60 identical DEGs were enriched in the GO terms, GO:0004601, GO:0016209, GO:0006979, GO:0072593, GO:0042744, and GO:0042743. Among these DEGs, 59 DEGs, including 23 upregulated and 36 downregulated DEGs, were annotated as POD, while 1 upregulated DEG was annotated as APX. Subsequently, we measured the H_2_O_2_ levels, POD activity, and APX activity in the roots of wheat seedlings. Under salt stress, H_2_O_2_ levels in root were significantly increased by 1.86-fold, whereas inoculation with WR10 significantly decreased the accumulation of H_2_O_2_ by 0.18-fold (Figure 4A). In contrast, APX activity significantly increased by 1.25-fold in the roots of WR10-inoculated seedlings compared to WR10-free plants under salt stress (Figure 4B). In addition, there was no difference in POD activity between the NaCl and NaCl + WR10 groups (Supplementary Figure 2).

### *Bacillus altitudinis* WR10 affects GSH metabolism and increases GSH levels under salt stress

**FIGURE 5:**
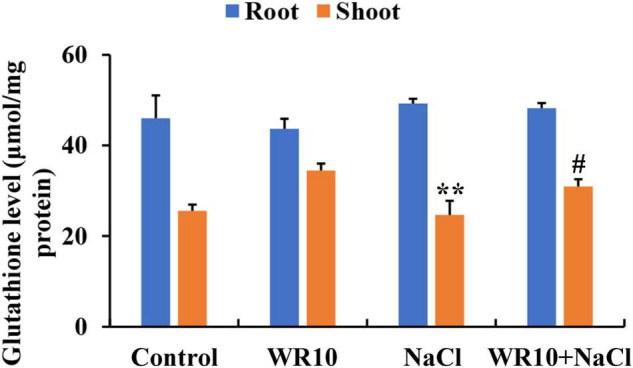
*Bacillus altitudinis* WR10 increases GSH levels in wheat seedlings under salt stress. Control, without NaCl and WR10; WR10, with WR10 and without NaCl; NaCl, with NaCl and without WR10; WR10 + NaCl, with NaCl and WR10. Data are expressed as the means ± SD (*n* = 6). **p < 0.01 (vs. control); ^#^*p* < 0.05 (vs. NaCl).

In the GSH metabolism pathway, WR10 inoculation significantly upregulated the expression levels of glutathione synthase (GSS), but significantly downregulated the expressions levels of glutathione S-transferase (GST) and gamma-glutamyltranspeptidase (GGT) under salt stress (Table 1). GSH levels in roots were not affected, but those in the shoots were significantly increased by 1.25-fold after inoculation with WR10 under salt stress (Figure 5).

**TABLE 1:** Identified DEGs related to GSH metabolism in NaCl vs WR10 + NaCl.

Gene ID	Description	FC (WR10 + NaCl/NaCl)	Log2FC (WR10 + NaCl/NaCl)	Regulate	*P* adjusted
**Glutathione metabolism**					
TraesCS7D02G431500	Glutathione synthase (GSS)	2.225	1.154	Up	0.004
TraesCS1A02G203900	Glutathione S-transferase (GST)	0.344	−1.540	Down	0.0001
TraesCS3D02G486800	GST	0.407	−1.298	Down	3.04E-09
TraesCS4B02G330800	GST	0.385	−1.375	Down	0.019
TraesCS4D02G327900	GST	0.351	−1.512	Down	0.0008
TraesCS7B02G398800	GST	0.435	−1.201	Down	0.004
TraesCS7D02G030800	GST	0.462	−1.115	Down	6.36E-07
TraesCS3B02G490400	GST	0.472	−1.083	Down	0.0009
TraesCS4A02G103800	GST	0.27	−1.888	Down	0.014
TraesCS2A02G045400	GST	0.352	−1.505	Down	7.65E-07
TraesCS1D02G190600	GST	0.449	−1.157	Down	0.001
TraesCS1B02G217700	GST	0.41	−1.287	Down	9.92E-05
TraesCS3B02G538600	GST	0.265	−1.913	Down	0.035
TraesCS4B02G199700	GST	0.374	−1.420	Down	0.0007
TraesCS4A02G103700	GST	0.452	−1.146	Down	0.011
TraesCS2D02G044100	GST	0.416	−1.267	Down	0.0001
TraesCS4D02G200700	GST	0.371	−1.429	Down	0.001
TraesCS5A02G502400	GST	0.266	−1.909	Down	0.0008
TraesCS1D02G207400	GST	0.462	−1.113	Down	0.001
TraesCS3A02G076500	Gamma-glutamyltranspeptidase (GGT)	0.429	−1.220	Down	0.010
TraesCS3B02G090400	GGT	0.329	−1.604	Down	2.00E-06

### *Bacillus altitudinis* WR10 activates phenylpropanoid biosynthesis under salt stress

**FIGURE 6:**
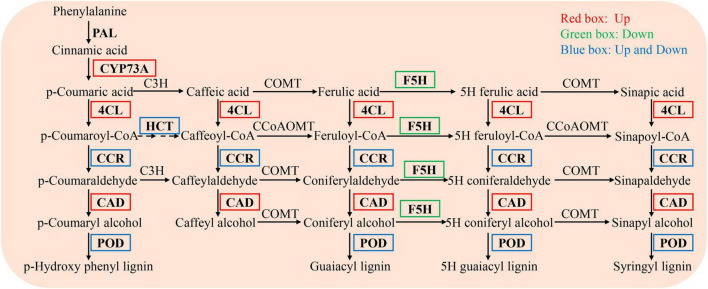
DEGs enriched in the phenylpropanoid biosynthesis pathway in the NaCl vs WR10 + NaCl. NaCl, with NaCl and without WR10; WR10 + NaCl, with NaCl and WR10. Red boxes, significantly up-regulated genes; Green boxes, significantly down-regulated genes; Blue boxes, significantly up- and downregulated genes.

In the phenylpropanoid biosynthesis pathway, the expression levels of trans-cinnamate 4-monooxygenase (CYP73A, EC 1.14.14.91), 4-coumarate-CoA ligase (4CL, EC 6.2.1.12), and cinnamyl alcohol dehydrogenase (CAD, EC 1.1.1.195) were significantly upregulated, while those of ferulate-5-hydroxylase (F5H, EC 1.14.-.-) were significantly downregulated. The levels of other genes, including shikimate O-hydroxycinnamoyltransferase (HCT, EC 2.3.1.133), cinnamoyl-CoA reductase (CCR, EC 1.2.1.44), and peroxidase (POD, EC 1.11.1.7), were either up- or downregulated (Figure 6).

### *Bacillus altitudinis* WR10 decreases proline content under salt stress

**FIGURE 7:**
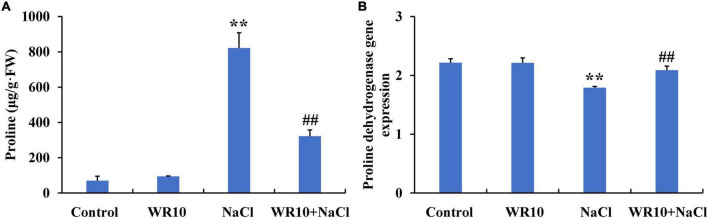
*Bacillus altitudinis* WR10 decreases the proline content **(A)** in the shoots and increases the proline dehydrogenase gene expression **(B)** in the roots of wheat seedlings under salt stress. Control, without NaCl and WR10; WR10, with WR10 and without NaCl; NaCl, with NaCl and without WR10; WR10 + NaCl, with NaCl and WR10. Data are expressed as the means ± SD (*n* = 6). **p < 0.01 (vs. control); ^##^*p* < 0.01 (vs. NaCl).

Under salt stress, proline content increased significantly by 11.7-fold in the shoots of wheat seedlings. In contrast, WR10 inoculation significantly reduced the proline content in salt-stressed wheat plants by 0.61-fold compared to the NaCl group without WR10 inoculation (Figure 7A). RNA-seq analysis showed that the gene expression levels of proline dehydrogenase (PDH) were significantly downregulated in salt-stressed wheat plants compared with those in the control group, but were significantly upregulated in WR10-inoculated plants under salt stress compared with those in the NaCl group without WR10 inoculation (Figure 7B). In addition, delta-1-pyrroline-5-carboxylate synthase (P5CS) and pyrroline-5-carboxylate reductase (P5CR), which are involved in proline biosynthesis, were not affected by WR10 inoculation (Supplementary Table 4).

## Discussion

Increased soil salinization seriously decreases the crop yield and endangers food security; it is one of the most threatening abiotic stresses. Wheat is moderately sensitive to salinity, and high salinity often results in growth delays and yield reduction (Royo and Abio, 2003). In this study, the growth parameters of wheat (cv. Zhoumai 36), including root length, shoot length, and root dry weight at the seedling stage, were inhibited via treatment with 150 mmol/L NaCl. This is consistent with other wheat varieties that are grown under salt stress (Quan et al., 2021). Many strategies have been developed to alleviate the adverse effects of salt stress in wheat. Use of plant-derived microbes to improve salt stress tolerance in wheat may be an excellent strategy because of the sustainability, eco-friendly nature, and economic significance of these microbes (Miransari and Smith, 2019). *B. altitudinis* WR10 is a salt-enduring endophyte that enhances the seed germination rate of wheat under salt stress (Yue et al., 2019). In this study, we investigated the effect of WR10 on the growth of salt-treated wheat seedlings and found that WR10 inoculation significantly improved the seedling growth in terms of root and shoot lengths and dry weights. This indicated that WR10 enhanced wheat salt tolerance at the seedling stage. Similar results were observed for plants inoculated with other *Bacillus* strains. For instance, *Bacillus methylotrophicus* M4-1-inoculated wheat shows increased plant height, root length, and dry weight under salt stress (Ji et al., 2020). Inoculation with *Bacillus licheniformis* HSW-16 significantly increases the root length, shoot length, and dry weight of salt-treated wheat (Singh and Jha, 2016b).

Generally, salt stress results in the accumulation of excess Na^+^ accompanied by a decrease in K^+^ levels and an imbalance in the Na^+^/K^+^ ratio, which is regarded as one of the main reasons for plant growth inhibition (Schachtman and Liu, 1999). In this study, salt-stressed wheat plants inoculated with WR10 showed lower Na^+^ and higher K + contents than uninoculated plants. In agreement with our results, a decrease in Na^+^ content and increase in K^+^ content was observed in wheat plants inoculated with *Bacillus velezensis* JC-K3 and *Klebsiella* sp. SBP-8 (Singh et al., 2015; Ji et al., 2021). We observed that two genes encoding plasma membrane H + -ATPase (AHA) were significantly upregulated in WR10-inoculated wheat roots under salt stress. Similar results were found in maize plants inoculated with *Serratia liquefaciens* KM4 (El-Esawi et al., 2018). Fan et al. (2018) revealed that *Arabidopsis* plants overexpressing AHA showed lower Na^+^ and higher K^+^ contents under salt stress. Therefore, WR10-induced upregulation of AHA expression may contribute to a lower Na^+^/K^+^ ratio in salt-stressed wheat. In addition to Na^+^ and K^+^, many studies have reported that PGPB also affects the uptake of other nutrients in plants under salt stress, such as P and Ca^2+^. In the present study, significant increases in P and Ca^2+^ levels were also found in salt-stressed wheat plants after WR10 inoculation. In a previous study, P-solubilizing bacteria *Bacillus* sp. L55, *Pseudomonas fluorescens* PMT1, *Pantoea* sp., and *Acinetobacter* sp. L176, which has phosphatase activity, has been reported to increase P uptake in maize and groundnut under salt stress conditions (Anzuay et al., 2017). WR10 was previously shown to have the ability to solubilize Ca_3_(PO4)_2_ and Ca-phytate because of its phosphatase and phytase activity (Yue et al., 2019). Therefore, the enhanced uptake of P could be closely associated with the P solubilizing characteristics of WR10. Another study reported an increased Ca^2+^ content in tomatoes under salt stress after *Glutamicibacter halophytocola* KLBMP 5180 inoculation. As a secondary messenger signaling molecule, Ca^2+^ plays an important role in plant growth, and its accumulation can increase plant resistance to salt stress (Yang et al., 2016). Collectively, these results show that WR10 may alleviate salt stress in wheat partly by improving Na^+^ exclusion and K^+^, P, and Ca^2+^ uptake.

To further explore the underlying mechanisms by which WR10 improves salt tolerance in wheat, transcriptome sequencing was performed. RNA-seq data showed that WR10 inoculation affected several DEGs involved in oxidative stress, ROS metabolism, and antioxidants under salt stress. It is well known that salt stress causes ROS accumulation and oxidative stress in plants (Tang et al., 2015). Some PGPB have been reported to alleviate salt stress by enhancing antioxidant enzymes. For instance, *B. licheniformis* promotes sunflower growth under saline conditions by increasing the expression of superoxide dismutase (SOD), catalase (CAT), and glutathione peroxidase (GPX) (Yasmeen et al., 2020). *Serratia marcescens* CDP-13 protects wheat plants from salt stress by enhancing the enzymes SOD, CAT, and POD (Singh and Jha, 2016a). Rice plants inoculated with *Bacillus pumilus* JPVS11 show an increase in SOD and CAT under salt stress (Kumar et al., 2021). Unlike these studies, no obvious changes in SOD, CAT, GPX, or POD were observed in our study. Notably, the gene expression and activity of APX were significantly increased in salt-treated wheat inoculated with WR10. APX is an important antioxidant enzyme in plant cells that plays a pivotal role in scavenging H_2_O_2_ (Sharma et al., 2012). Overexpression of APX in tomatoes and rice enhances APX activity and leads to a reduction in H_2_O_2_ under salt stress (Wang et al., 2005; Zhang et al., 2013). Therefore, WR10 may reduce H_2_O_2_ levels under salt conditions via the activation of APX. Indeed, we observed a decrease in H_2_O_2_ levels in the roots of salt-treated wheat plants after inoculation with WR10.

In addition to enzymatic processes, plants produce many non-enzymatic components to withstand oxidative stress. GSH is a crucial antioxidant for ROS scavenging including H_2_O_2_ (Gill and Tuteja, 2010). In this study, increased GSH levels, upregulated GSS, and downregulated GST and GGT were found in WR10-inoculated plants under salt stress. GSS is a key enzyme responsible for GSH synthesis and catalyzes the formation of GSH from γGC and glycine (Ogawa, 2005). GST and GGT are important enzymes involved in GSH metabolism, in which GST catalyzes the conjugation of GSH to electrophilic xenobiotic substrates and GGT is responsible for GSH degradation (Labrou et al., 2015; Bachhawat and Kaur, 2017). Therefore, the increase in GSH synthesis and decrease in GSH metabolism induced by WR10 led to an increase in GSH levels in salt-treated wheat. Similarly, increased GSH levels were observed in salt-stressed pea plants after inoculation with *B. subtilis* and *P. fluorescens* (Sofy et al., 2021). We suggest that WR10 may accelerate the removal of H_2_O_2_ by elevating GSH levels.

KEGG enrichment analysis showed that WR10 inoculation affected many genes involved in phenylpropanoid biosynthesis under salt stress, indicating that this pathway may play an important role in alleviating the effects of WR10 on salt stress. The phenylpropanoid pathway is one of the most important metabolic pathways in plants and produces more than 8000 metabolites (Zhang and Liu, 2015). In this pathway, phenylalanine ammonia lyase (PAL) is the initial enzyme that catalyzes the formation of trans-cinnamic acid from phenylalanine. Cinnamic acid is then hydroxylated by cinnamic acid 4-hydroxylase (CYP73A) and the resultant p-coumaric acid is further catalyzed by 4-coumarate-CoA ligase (4CL) to generate p-coumaroyl-CoA (Dong and Lin, 2021). In our study, significantly upregulated CYP73A and 4CL were found in salt-stressed plants after inoculation with WR10, indicating that WR10 may increase the p-coumaroyl-CoA content. p-coumaroyl-CoA is important precursor for all downstream metabolites in the phenylpropanoid pathway, including phenolics and flavonoids (Vogt, 2010; Sharma et al., 2019). This increase may provide more substrate for these compounds protecting plants against abiotic stress. Li et al. (2021) indicated that N deficiency induces phenolic and flavonoid accumulation by upregulating the gene expression of PAL, CYP73A, and 4CL in *Coreopsis tinctoria*. Kim et al. (2014) reported that overexpression of CYP73A and 4CL promotes flavone accumulation in *Scutellaria baicalensis* roots. Therefore, activation of the phenylpropanoid pathway may be another strategy for WR10 to improve the salt tolerance of wheat.

Proline is an important osmolyte in plants, and its accumulation often occurs when plants are exposed to abiotic stress (Hayat et al., 2012). Many plants, including wheat, produce high levels of proline under salt stress. Surprisingly, WR10-inoculation resulted in significantly lower proline levels in salt-stressed wheat plants. Similar results were observed in *Pseudomonas azotoformans* CHB 1107-inoculated tomato plants and *Azotobacter chroococcum*-inoculated maize plants under salt stress (Rojas-Tapias et al., 2012; Liu et al., 2021). The proline content is controlled by proline biosynthesis and degradation. In this study, the gene expression of P5CS and P5CR related to proline biosynthesis was not affected by WR10 inoculation under salt stress, whereas PDH mRNA levels were significantly upregulated. PDH is a key enzyme involved in proline degradation, and degrades proline to P5C (Wang et al., 2015). Therefore, we speculated that upregulated PDH expression contributed to the reduction of proline.

In addition to affecting plants, our previous study found that WR10 relieved the adverse effects of salt stress on wheat seed germination through its inherent tolerance to NaCl, biofilm formation, and production of ACC deaminase (Yue et al., 2019). Biofilms enhance bacterial surface attachment in roots and contain many exopolysaccharides (EPS), which chelate dissociated Na^+^ and restrict Na^+^ importation into the roots (Ashraf et al., 2004; Dodd and Pérez-Alfocea, 2012). ACC deaminase is an endogenous enzyme in endophytes that catalyzes the conversion of ACC, a precursor of ethylene biosynthesis, into ammonia and α-ketobutyric acid. Many ACC deaminase-producing bacteria can reduce plant-produced ACC, thereby inhibiting ethylene production and mitigating salt stress in plants (Misra and Chauhan, 2020; Orozco-Mosqueda et al., 2020). Therefore, we believe that the salinity-alleviating properties of WR10 in wheat can also be attributed to the biofilm and ACC deaminase produced by WR10.

## Conclusion

In conclusion, this study confirmed that the halotolerant strain, *B. altitudinis* WR10, improved the salt tolerance of wheat at the seedling stage. Therefore, this endophyte has the potential to improve crop growth under high-salt stress as a novel bioinoculant. Furthermore, we revealed that the mitigating effects of WR10 can be attributed to the following aspects: (1) its inherent plant growth-promoting traits and salt resistance; (2) improved Na^+^/K^+^ homeostasis and increased uptake of nutrients such as P and Ca^2+^; (3) enhanced H_2_O_2_ scavenging via increased APX activity and GSH levels; and (4) activation of the phenylpropanoid pathway. These findings provide a theoretical basis for the application of WR10 in saline soil. However, further studies are necessary to verify the salinity-alleviating properties of WR10 as a bioinoculant in actual fields, considering the complexity of the soil environment in these fields.

## Data Availability Statement

The datasets presented in this study can be found in online repositories. The names of the repository/repositories and accession number(s) can be found in the article/Supplementary Material.

## Author contributions

ZY designed the experiments and wrote the draft manuscript. YW, LZ, QZ, YL, and CH performed the experiments. YC and KM analyzed the results. CC uploaded RNA-seq data. ZS provided the raw materials and financial support and completed the final manuscript. All authors have read and approved the manuscript.

## Conflict of Interest

The authors declare that the research was conducted in the absence of any commercial or financial relationships that could be construed as a potential conflict of interest.

## Publisher’s Note

All claims expressed in this article are solely those of the authors and do not necessarily represent those of their affiliated organizations, or those of the publisher, the editors and the reviewers. Any product that may be evaluated in this article, or claim that may be made by its manufacturer, is not guaranteed or endorsed by the publisher.
