# Supplementary material for: Halotolerant Bacillus altitudinis WR10 improves salt tolerance in wheat via a multi-level mechanism
Source: Front Plant Sci. 2022 Jul 14;13:941388. doi: 10.3389/fpls.2022.941388 (PMC9330482; doi:10.3389/fpls.2022.941388)
Supplement: Supplementary file 1 [file Image_1.PDF]

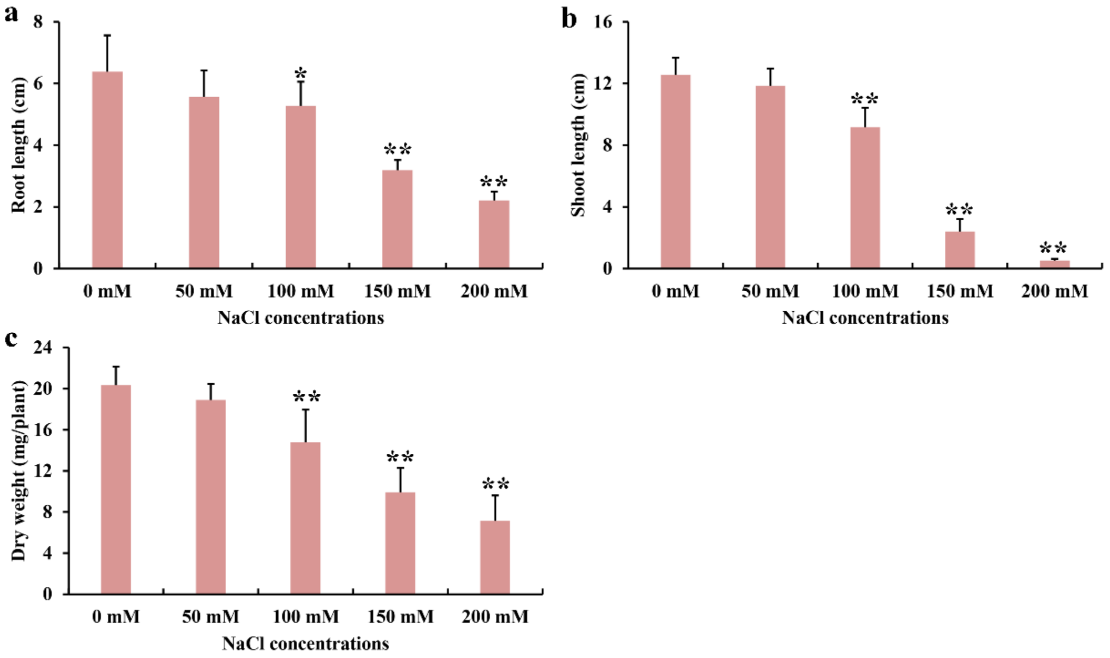

FIGURE S1 The effect of NaCl on wheat growth. (A) Root length; **(B)** Shoot length; **(C)** Plant dry weight. Seeds of Zhoumai 36 were grown in petri dishes containing 50-200 mM NaCl for 1 week.
