# Supplementary material for: Halotolerant Bacillus altitudinis WR10 improves salt tolerance in wheat via a multi-level mechanism
Source: Front Plant Sci. 2022 Jul 14;13:941388. doi: 10.3389/fpls.2022.941388 (PMC9330482; doi:10.3389/fpls.2022.941388)
Supplement: Supplementary file 2 [file Image_2.PDF]

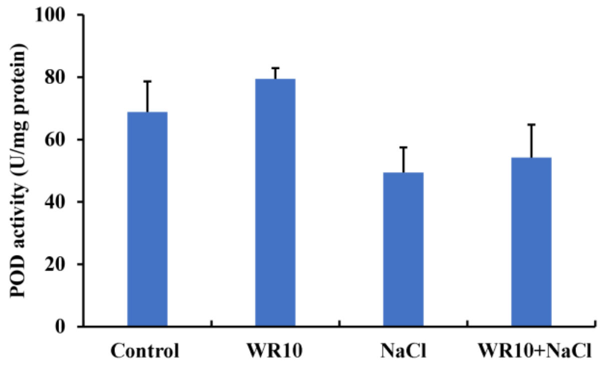

FIGURE S2 POD activity in root of wheat seedlings with / without *Bacillus altitudinis* WR10 under salt stress.
