# Supplementary material for: Halotolerant Bacillus altitudinis WR10 improves salt tolerance in wheat via a multi-level mechanism
Source: Front Plant Sci. 2022 Jul 14;13:941388. doi: 10.3389/fpls.2022.941388 (PMC9330482; doi:10.3389/fpls.2022.941388)
Supplement: Supplementary file 3 [file Table_1.DOCX]

**Table S1** Summary of the RNA-Seq data in wheat roots with / without *Bacillus altitudinis* WR10 under salt stress

| **Sample** | **Raw reads** | **Clean reads** | **Q20 (%)** | **Q30 (%)** | **GC content (%)** |
| --- | --- | --- | --- | --- | --- |
| Control 1 | 63,525,054 | 62,681,842 | 98.22 | 94.55 | 52.86 |
| Control 2 | 70,396,280 | 69,655,306 | 98.22 | 94.57 | 54.72 |
| Control 3 | 72,964,816 | 72,071,690 | 98.22 | 94.6 | 54.37 |
| NaCl 1 | 75,743,832 | 75,082,610 | 98.31 | 94.79 | 54.98 |
| NaCl 2 | 73,272,960 | 72,562,582 | 98.21 | 94.56 | 55.19 |
| NaCl 3 | 64,861,102 | 64,160,712 | 98.15 | 94.41 | 55.1 |
| WR10 1 | 63,506,984 | 62,696,142 | 98.16 | 94.44 | 54.25 |
| WR10 2 | 64,866,266 | 64,189,140 | 98.05 | 94.14 | 54.19 |
| WR10 3 | 65,126,876 | 64,326,858 | 98.22 | 94.59 | 53.4 |
| WR10+NaCl 1 | 64,938,234 | 64,302,240 | 98.15 | 94.39 | 54.69 |
| WR10+NaCl 2 | 63,722,920 | 63,034,532 | 98.11 | 94.3 | 55.16 |
| WR10+NaCl 3 | 64,697,918 | 63,968,036 | 98.16 | 94.47 | 55.2 |
| Total | 807,623,242 | 798,731,690 |  |  |  |
| Average |  |  | 98.18 | 94.48 | 54.51 |
