# Supplementary material for: Halotolerant Bacillus altitudinis WR10 improves salt tolerance in wheat via a multi-level mechanism
Source: Front Plant Sci. 2022 Jul 14;13:941388. doi: 10.3389/fpls.2022.941388 (PMC9330482; doi:10.3389/fpls.2022.941388)
Supplement: Supplementary file 4 [file Table_2.DOCX]

**Table S2** Statistics of alignment results

| **Sample** | **Total mapped** | **Multiple mapped** | **Uniquely mapped** |
| --- | --- | --- | --- |
| Control 1 | 52,105,866 (83.13%) | 3,513,447 (5.61%) | 48,592,419 (77.52%) |
| Control 2 | 57,068,948 (81.93%) | 3,462,755 (4.97%) | 53,606,193 (76.96%) |
| Control 3 | 56,753,564 (78.75%) | 3,543,416 (4.92%) | 53,210,148 (73.83%) |
| NaCl 1 | 61,590,751 (82.03%) | 3,844,402 (5.12%) | 57,746,349 (76.91%) |
| NaCl 2 | 59,034,940 (81.36%) | 3,760,678 (5.18%) | 55,274,262 (76.17%) |
| NaCl 3 | 53,231,824 (82.97%) | 3,293,321 (5.13%) | 49,938,503 (77.83%) |
| WR10 1 | 49,400,689 (78.79%) | 3,234,294 (5.16%) | 46,166,395 (73.64%) |
| WR10 2 | 52,513,242 (81.81%) | 3,223,229 (5.02%) | 49,290,013 (76.79%) |
| WR10 3 | 53,532,458 (83.22%) | 3,638,437 (5.66%) | 49,894,021 (77.56%) |
| WR10+NaCl 1 | 51,241,659 (79.69%) | 3,294,905 (5.12%) | 47,946,754 (74.56%) |
| WR10+NaCl 2 | 50,698,619 (80.43%) | 3,380,414 (5.36%) | 47,318,205 (75.07%) |
| WR10+NaCl 3 | 50,374,798 (78.75%) | 3,337,994 (5.22%) | 47,036,804 (73.53%) |
