# Supplementary material for: Halotolerant Bacillus altitudinis WR10 improves salt tolerance in wheat via a multi-level mechanism
Source: Front Plant Sci. 2022 Jul 14;13:941388. doi: 10.3389/fpls.2022.941388 (PMC9330482; doi:10.3389/fpls.2022.941388)
Supplement: Supplementary file 5 [file Table_3.DOCX]

**Table S3** Summary of the gene annotations in six databases

| Data base | gene number(percent) | transcript number(percent) |
| --- | --- | --- |
| GO | 72,653 (83.72%) | 89,126 (84.36%) |
| KEGG | 31,075 (35.81%) | 40,288 (38.13%) |
| COG | 81,038 (93.38%) | 99,696 (94.36%) |
| NR | 86,457 (99.63%) | 105,355 (99.72%) |
| Swiss-Prot | 66,318 (76.42%) | 82,780 (78.35%) |
| Pfam | 72,170 (83.16%) | 89,131 (84.36%) |
| Total annotations | 86,538 (99.72%) | 105,438 (99.8%) |
| Total | 86,781 (100%) | 105,650 (100%) |

Note: GO, Gene Ontology; KEGG, Kyoto Encyclopedia of Genes and Genomes; COG, Clusters of Orthologous Groups of proteins database; NR, NCBI non-redundant protein database; Swiss-Prot, reviewed protein sequence database.
