# Supplementary material for: Halotolerant Bacillus altitudinis WR10 improves salt tolerance in wheat via a multi-level mechanism
Source: Front Plant Sci. 2022 Jul 14;13:941388. doi: 10.3389/fpls.2022.941388 (PMC9330482; doi:10.3389/fpls.2022.941388)
Supplement: Supplementary file 6 [file Table_4.DOCX]

**Table S4** The gene expression related to GSH biosynthesis in NaCl vs WR10+NaCl

| **Gene ID** | **Description** | **Log2FC (WR10+NaCl/NaCl)** | **p-adjusted** |
| --- | --- | --- | --- |
| TraesCS1D02G280700 | Delta-1-pyrroline-5-carboxylate synthase (P5CS) | -0.06 | 0.865 |
| TraesCS1B02G290600 | P5CS | -0.219 | 0.356 |
| TraesCS3B02G538100 | Pyrroline-5-carboxylate reductase (P5CR) | -0.400 | 0.268 |
| TraesCS3D02G483400 | P5CR | -0.1801 | 0.773 |
